# Relationship of hemoglobin levels and distribution and severity of gingival melanin pigmentation: An exploratory cross-sectional study

**DOI:** 10.34172/japid.2024.020

**Published:** 2024-09-11

**Authors:** Riya Achamma Daniel, Veena HR, Suman Basavaraju

**Affiliations:** ^1^Department of Dental Surgery, MIOT International, Chennai, India; ^2^Department of Periodontics, K. L. E. Society’s Institute of Dental Sciences, Bangalore, India; ^3^Department of Periodontics, JSS Dental College and Hospital, JSS Academy of Higher Education and Research, Mysuru, India

**Keywords:** Gingiva, Hemoglobin, Melanin, Pigmentation

## Abstract

**Background.:**

Recurrence of gingival pigmentation following depigmentation procedures is common, raising the question of the influence of an underlying cause, if any. Melanin, a non-hemoglobin-derived brown pigment, is the most common endogenous pigment contributing to gingival pigmentation. Hemoglobin derivatives are among the other prime pigments that contribute to gingival color. This exploratory cross-sectional study evaluated the influence of hemoglobin levels on the distribution and severity of gingival melanin pigmentation in periodontitis, gingivitis, and healthy periodontium.

**Methods.:**

Twenty subjects with periodontitis (group 1), gingivitis (group 2), and healthy periodontium (group 3) were recruited in this observational study, totaling 60 subjects. The hemoglobin levels in g/dL and Hedin Melanin Index (HMI-1977) scores were recorded for all subjects, and relevant statistical tests were applied (*P*<0.05).

**Results.:**

A negative correlation was observed between the hemoglobin levels and the HMI scores for the whole sample and each group. This correlation was statistically significant for the whole sample and for the gingivitis group in which the correlation was moderate.

**Conclusion.:**

The inverse and significant relation between the hemoglobin levels and distribution and severity of melanin pigmentation observed for the whole sample and the gingivitis group requires further research and validation to identify and manage the confounding factors in the treatment of gingival pigmentation.

## Introduction

 Harmony of an esthetic smile requires perfect integration of facial composition that considers both hard and soft tissues of the face and dental composition, which more specifically includes the teeth and their relationship to gingival tissues. The health and appearance of gingiva are essential components of an attractive smile.

 The gingiva is the most frequently physiologically pigmented tissue in the oral cavity. The color of the gingiva varies between individuals and is thought to be associated with cutaneous pigmentation.^1^ The gingival color primarily depends upon the quantity and quality of vasculature, epithelial thickness, degree of keratinization, and pigments within the gingival epithelium. Pathological gingival pigmentation is a discoloration of the gingiva due to a variety of lesions and conditions associated with various endogenous and exogenous etiologic agents.^2^

 Melanin, a non-hemoglobin-derived brown pigment, is the most common of the endogenous pigments and is produced by melanocytes in the basal and suprabasal cell layers of the epithelium.^3^ Hemoglobin derivatives, including oxyhemoglobin, reduced hemoglobin, bilirubin, and biliverdin, are among the other prime pigments contributing to gingival pigmentation.^4,5^ The results of a previous in vivo study suggest the influence of hemoglobin on gingival pigmentation in children.^6^ Furthermore, a linear transformation was observed of erythema Δa* and brightness ΔL* into ΔHemoglobin = 1.68Δa* + 0.60ΔL* and ΔMelanin = -1.06a* - 1.44ΔL*.^7^ However, there has been no sufficient evidence in the available literature associating hemoglobin levels and the melanin pigmentation of gingiva in the adult human population. Recurrence of gingival pigmentation following depigmentation procedures is common and raises the question of the influence of an underlying cause, if any.

 Thus, this exploratory cross-sectional study evaluated the influence of hemoglobin levels on the distribution and severity of gingival melanin pigmentation in periodontitis, gingivitis, and healthy periodontium.

## Methods

 The study population consisted of 60 volunteers, in whom routine blood investigations were indicated before dental treatment. Following clearance from the institutional ethics committee and registration with the Clinical Trials Registry India, the subjects were recruited by simple random sampling from the out-patient pool of a tertiary dental hospital in South India after providing each of them with an information sheet, explaining the purpose, nature, and design of the study and obtaining written informed consent.

 The sample size was estimated using the software G*Power v. 3.1.9.2. Considering the effect size to be measured (f) at 40%, the power of the study at 80%, and the margin of the α-error at 5%, the total sample size was estimated at n = 60 (20 in each group).

 Following routine periodontal examination, systemically healthy males and females 20‒60 years of age with no signs of pathological gingival pigmentation were categorized into three groups based on case definitions following the official proceedings from the 2017 World Workshop on the Classification of Periodontal and Peri-Implant Diseases and Conditions, in association with American Academy of Periodontology and the European Federation of Periodontology.

- Group 1: periodontitis
- Group 2: gingivitis
- Group 3: healthy periodontium

The criteria for exclusion included any signs of pathological gingival pigmentation, including pigmentation from chronic consumption of certain drugs, systemic diseases and syndromes, and neoplasms of the gingiva.^8^

**Figure 1:**
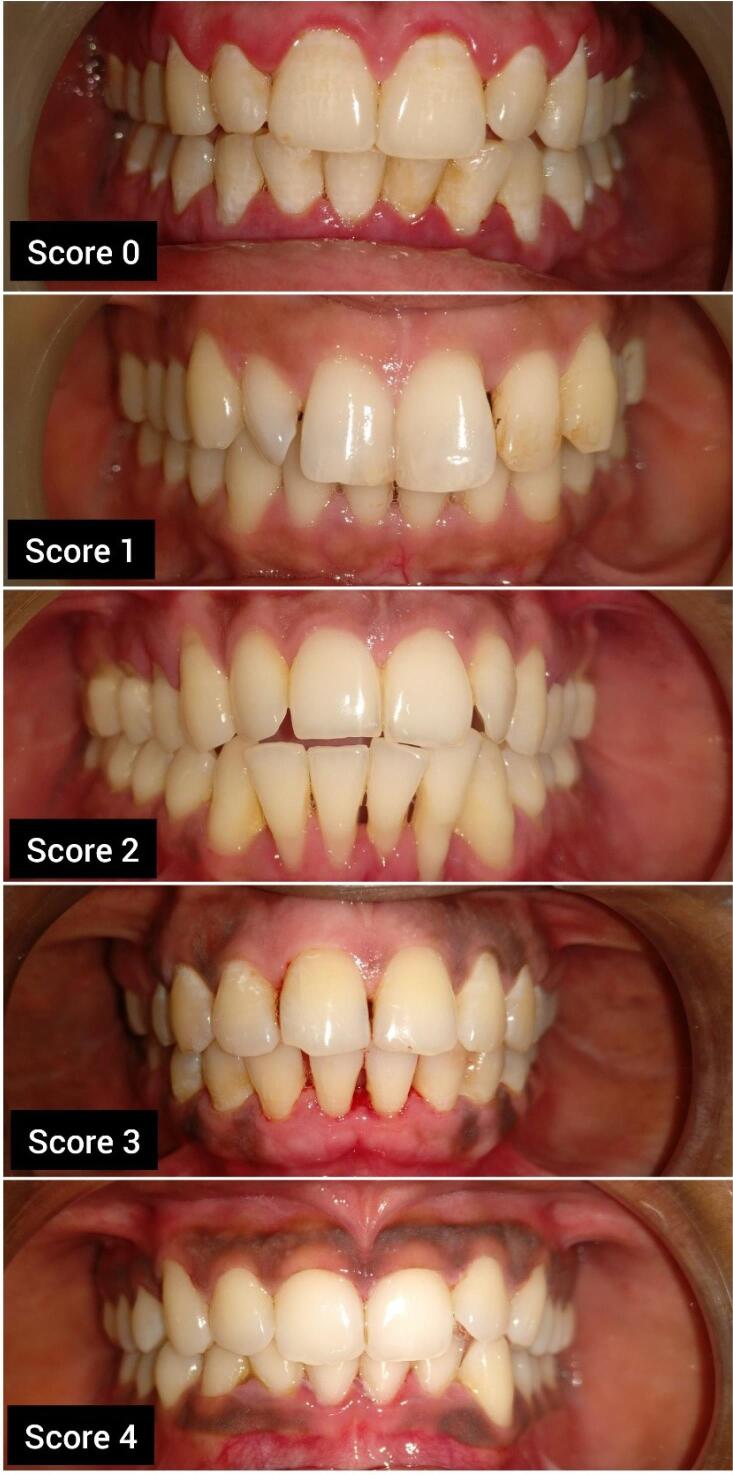
Hedin melanin index

 The severity and distribution of the melanin pigmentation were assessed using the Hedin Melanin Index (HMI) (1977)^8^ as follows (Figure 1):

- Score 0: no pigmentation
- Score 1: one or two solitary unit(s) of pigmentation in papillary gingiva without any continuous ribbon
- Score 2: more than three units of pigmentation in papillary gingiva without a continuous ribbon of pigmentation
- Score 3: one or more short continuous pigmented ribbons
- Score 4: One continuous ribbon spreading over the entire area between canines
- The levels of hemoglobin in the blood in g/dL were recorded employing Sahli’s acid haematin method.

 Statistical Package for Social Sciences SPSS for Windows, version 22.0. released in 2013 (Armonk, NY: IBM Corp.) was used to perform statistical analyses. Descriptive analysis included the expression of outcome variables in terms of mean and standard deviation for continuous variables, with frequency and proportions for categorical variables. The normality of the data was verified using the Shapiro-Wilk test, which showed that the data did not follow a normal distribution. Hence, all the inferential statistical tests were performed using relevant non-parametric tests. The level of significance was set at *P* < 0.05.

## Results

 The mean age in group 1 was 34.7 years, with 33.2 and 35.3 years in groups 2 and 3, respectively. Group 1 showed a higher male composition, while groups 2 and 3 showed a higher female composition. However, there were no significant intergroup differences in the demographic data (Table 1).

**Table 1:** Distribution of demographic characteristics and study variables in study subjects

**Variable**	**Category**	**Group 1 (n=20)**		**Group 2 (n=20)**		**Group 3 (n=20)**		***P*** **-value**
		**Mean**	**SD**	**Mean**	**SD**	**Mean**	**SD**	
Age	Mean and SD	34.7	7.5	33.2	7.7	35.3	11.6	0.82^a^
	Range	20 – 48		20 – 48		20 - 57		
		**n**	**%**	**n**	**%**	**n**	**%**	
Gender	Males	13	65%	6	30%	7	35%	0.06^b^
	Females	7	35%	14	70%	13	65%	
		**Mean**	**SD**	**Mean**	**SD**	**Mean**	**SD**	
Hemoglobin		13.30	1.90	12.33	1.93	12.93	1.97	0.38^a^
	Males	14.62	0.51	15.03	0.60	15.41	0.65	
	Females	10.84	0.28	11.16	0.70	11.59	0.61	
	*P* value	< 0.001*^c^		< 0.001*^c^		< 0.001*^c^		
HMI		2.90	1.02	2.70	1.22	2.05	1.32	0.09^a^
	Males	2.85	1.14	2.83	1.17	2.29	1.70	
	Females	3.00	0.82	2.64	1.28	1.92	1.12	
	*P* value	0.94^c^		0.84^c^		0.54^c^		

^a^ Kruskal Wallis test; ^b^ chi-squared test; ^c^ Mann-Whitney test. * Statistically significant.

 The mean hemoglobin was the highest in group 1 and the least in group 2, but the intergroup difference was not statistically significant (Table 1). The gender-wise distribution of the mean hemoglobin levels showed significantly higher values in males compared to females in all the three groups (Table 1). The highest mean hemoglobin values were found in group 3, followed by groups 2 and 1, with a statistically significant intergroup difference for both males and females. The pair-wise comparison between groups 1 and 3 was also significant (Table 2).

**Table 2:** Comparison of mean hemoglobin levels and mean HMI scores between different groups in males and females using the Kruskal-Wallis test followed by Post hoc Mann-Whitney test

**Parameter**	**Gender**	**Groups**	**N**	**Mean**	**SD**	***P*** **value**^a^	**Sig. Diff**	***P*** **value**^b^
Hemoglobin	Males	Group 1	13	14.62	0.51	0.04*	G1 Vs G2	0.18
		Group 2	6	15.03	0.60		G1 Vs G3	0.02*
		Group 3	7	15.41	0.65		G2 Vs G3	0.25
	Females	Group 1	7	10.84	0.28	0.008*	G1 Vs G2	0.06
		Group 2	14	11.16	0.70		G1 Vs G3	0.004*
		Group 3	13	11.59	0.61		G2 Vs G3	0.07
HMI scores	Males	Group 1	13	2.85	1.14	0.82	G1 Vs G2	-
		Group 2	6	2.83	1.17		G1 Vs G3	-
		Group 3	7	2.29	1.70		G2 Vs G3	-
	Females	Group 1	7	3.00	0.82	0.10	G1 Vs G2	-
		Group 2	14	2.64	1.28		G1 Vs G3	-
		Group 3	13	1.92	1.12		G2 Vs G3	-

* Statistically significant.

 The mean HMI scores were highest in group 1, followed by groups 2 and 3, with no significant intergroup difference (Table 1). The difference in mean HMI scores between the males and females was not statistically significant in any of the groups (Table 1). For both males and females, the highest mean HMI scores were found for group 1 and the least for group 3, although there was no significant difference in intergroup analysis and pair-wise comparison (Table 2).

 The relationship between hemoglobin and HMI scores in the total sample showed a weak negative correlation of statistical significance. This relationship in the three groups was also negative; however, while group 2 alone showed a moderate negative correlation of statistical significance, groups 1 and 3 showed a weak negative correlation (Table 3).

**Table 3:** Relationship between Hemoglobin levels and HMI score among study subjects

**Samples**	**Variable**	**Values**	**HMI Score**
Total Sample	Hb	Rho	-0.31
		*P* value	0.02*
		N	60
Group 1	Hb	Rho	-0.37
		*P* value	0.11
		N	20
Group 2	Hb	Rho	-0.49
		*P* value	0.03*
		N	20
Group 3	Hb	Rho	-0.33
		*P* value	0.15
		N	20

* Statistically significant. Spearman’s correlation test. The correlation coefficients are denoted by ‘rho’. The minus sign denotes a negative correlation. Correlation coefficient range: – no correlation; 0.01-0.20 – very weak correlation; 0.21-0.40 – weak correlation; 0.41-0.60 - moderate correlation; 0.61-0.80 – strong correlation; 0.81-1.00 – very strong correlation.

**Figure 2:**
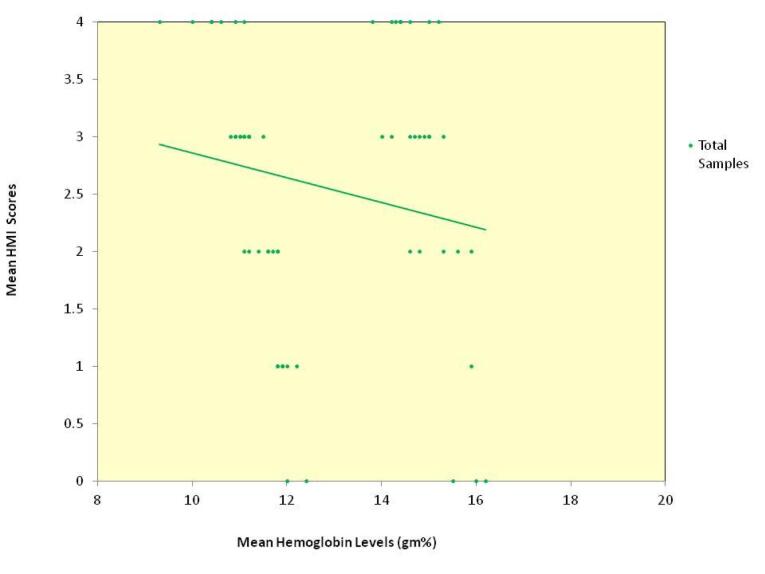
Scatter plot depicting the relationship between mean hemoglobin levels and mean HMI scores in the total sample

**Figure 3:**
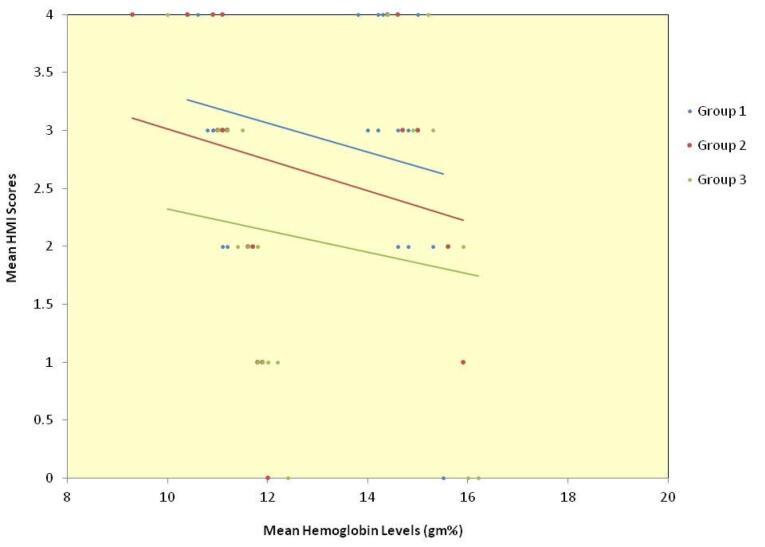
Scatter plot depicting the relationship between mean hemoglobin levels and mean HMI scores for each of the three groups

 The relationship between age and hemoglobin levels was a weak negative correlation of statistical significance for the whole sample. Among the groups, group 2 alone showed a statistically significant moderate correlation, group 1 showed a very weak negative correlation, and group 3 showed a weak negative correlation (data not presented). The relationship between the age and the HMI scores for the whole sample and groups 1 and 3 individually showed a very weak negative correlation, and group 2 showed weak negative correlation, none of which were statistically significant (data not presented). The scatter plot depicting the relationship between mean hemoglobin levels and mean HMI scores in the total sample (Figure 2) and for each of the three groups (Figure 3) showed an inverse relationship.

## Discussion

 The beauty in a smile can be traced back to the earliest civilizations. The Phoenicians (800 BC) and Etruscans (900 BC) carefully carved animal tusks to simulate the natural shape, form, and hue of teeth. Cosmetic expectations have increased with time, and in this era of pink and white esthetics, current trends speak volumes about gingival esthetics and smile designing. Gingiva is physiologically coral pink or salmon pink in color, with physiological pigmentation variations, especially melanin.^9^ Dummett and Bolden^10^ suggested that the degree of pigmentation depends on many factors, including chemical, mechanical, and physical stimulation. However, Stokowski et al.^11^ reported a significant association between cutaneous pigmentation and genetics.

 Melanin, a non-hemoglobin-derived brown pigment produced by melanocytes, is the most common endogenous pigment in the gingiva, especially in dark-skinned individuals. A clinician may recommend a depigmentation procedure when the patient scores 1‒2 of gingival melanin pigmentation and pigmented lesionsindex proposed by Peeran et al^8^ and has up to a class 2 of Liebart and Deruelle smile line classification.^8^ Pigmentation recurrence post-treatment is common to occur as early as 24 hours to as late as 8 years.^12^ However, Dummett^13^ noted that certain dark-complexioned individuals possessed perfectly pink gums devoid of any melanogenic pigmentation, raising questions about any underlying masked etiology in gingival pigmentation apart from racial differences in the absence of any state of pathology.

 In this study, the demographic data showed no statistically significant difference between the three groups for both age-wise and gender-wise comparisons. Our finding of a large margin of difference in women’s composition between the periodontitis group compared to gingivitis and healthy groups may be attributed to the fact that women usually care more about their body and appearance and, therefore, may be more concerned about adopting behaviors and habits, which promote their dental health.^14^

 Upon gender stratification, there was a statistically significant difference in the mean hemoglobin levels between the three groups and between periodontitis and healthy periodontium groups. Similar findings were observed in a study where periodontitis subjects showed a significantly lower hemoglobin level than gingivitis and healthy subjects.^15^ The intragroup comparison of mean hemoglobin levels in each of the three groups showed significantly higher values in males than females, a fact universally accepted and attributed to the physiological phenomenon of the direct effect of sex hormones, estrogen, and androgens on erythropoiesis.^16^ However, the mean values were much below the normal hemoglobin range in the women of all the three groups, which may be attributed to nutritional anemia resulting from micronutrient deficiency being prevalent in more than 50% of women in India.^17^ The relationship between age and hemoglobin showed a negative correlation, which was statistically significant for the overall sample in accordance with the observation made by Hawkins et al.^18^

 Despite having observed extreme non-pigmented scores as well, mean HMI scores in this study were to the higher end, attributed to the pigmented nature of Asians in accordance with a study conducted among South Indian subjects.^19^ The intergroup comparison of mean HMI scores before and after gender stratification in this study showed no significant difference. However, the scores were the highest in the periodontitis group and lowest in the healthy group, while females recorded the lower scores in groups except for periodontitis. However, when attempting to associate with age, HMI scores showed a negative correlation between the whole sample and each of the groups in the study.

 The relationship between the hemoglobin levels and HMI scores revealed a negative correlation, which was statistically significant for the whole sample and the gingivitis group. This is consistent with an in vivo study conducted in 83 children, which suggested an influence of hemoglobin concentration on gingival pigmentation. At lower hemoglobin concentrations in children, a significantly higher level of gingival pigmentation score was observed compared to higher hemoglobin concentrations.^6^ In an in vivo study on 15 Japanese subjects, hemoglobin and melanin were believed to be respective determinants of erythema (a*) and brightness (L*). The results of a graphic analysis indicated a linear transformation of Δa* and ΔL* into (ΔHb = 1.68Δa* + 0.60ΔL*) and (ΔMel = -1.06a* - 1.44ΔL*).^7^

 This association, however, has no sufficient evidence in the literature to establish a statement. Biochemically, L-DOPA, the direct precursor of dopamine, is synthesized from L-tyrosine by the enzyme tyrosine hydroxylase with tetrahydrobiopterin and iron as cofactors in the melanin synthesis pathway.^20^ Moreover, in an animal model using rats, nutritional iron deficiency induced the reduction of dopamine receptor binding sites, resulting in the down-regulation of dopaminergic activity similar to that observed in antipsychotic-treated animals.^21^ Thus, iron deficiency can impair the synthesis of dopamine.^22^ Therefore, further studies are required to identify an accumulation of tyrosine, if any, with decreased hemoglobin levels from a deficient serum iron level that can alter this pathway and redirect it toward melanin synthesis. The statistically non-significant results observed in this study might be attributed to the limitations of this study, including the small sample size, the application of Spearman’s correlation test without categorizing the genders, and the application of an index rather than quantifying the local distribution of melanin in the gingiva.

 A weak negative correlation of statistical significance between the mean hemoglobin levels in g/dL and the mean HMI scores of 1977 for the total sample was observed. A moderate correlation of statistical significance was found in the gingivitis group. Upon gender stratification, intergroup comparison of mean hemoglobin levels showed a decreasing trend from periodontal health to periodontitis. On gender categorization, the mean HMI scores decreased from periodontitis to periodontal health. While these observations pave the way for confounding factors that may unravel the science behind the recurrence of pigmentation following gingival depigmentation, the grounds for these associations remain elusive. This necessitates further studies involving a larger sample to validate and understand the inferences from this exploratory research and employ them to manage gingival pigmentation in the absence of other pathological etiological factors.

## Acknowledgments

 The authors would like to acknowledge the late Prof. Dr. Sudhir R. Patil for laying the foundation for this research.

## Competing Interests

 The authors declare no competing interests.

## Consent for Publication

 Not applicable.

## Data Availability Statement

 Not applicable.

## Ethical Approval

 Volunteering individuals with written informed consent were recruited into the study after approval of the Institutional Ethics Committee. This study was approved by the Institutional Ethics Committee (IEC) of KLE Society’s Institute of Dental Sciences (IEC No: KIDS/IEC/NOV-19/38) and registered with the Clinical Trials Registry India (CTRI Registration number: CTRI/2019/12/022434).
